# Multi-omics analysis of flavor differences in pectoral muscles between Wuqin 10 duck and Cherry valley duck

**DOI:** 10.3389/fmolb.2025.1558907

**Published:** 2025-05-30

**Authors:** Ping Gong, Shengqiang Ye, Xing Chen, Lixia Wang, Yunguo Qian, Mingli Zhai, Yu Yang

**Affiliations:** Animal Husbandry and Veterinary Research Institute, Wuhan Academy of Agricultural Sciences, Wuhan, Hubei, China

**Keywords:** duck, pectoral muscles, metabolomics, lipidomics, transcriptomics, flavor substances

## Abstract

This study aimed to explore the impact of breeds on the lipid composition and flavor substances in duck pectoral muscles. In this study, 63-day-old Wuqin 10 ducks (WQ) and Cherry Valley ducks (CV) were selected as the research objects. The pectoral muscle tissues were collected. The composition of volatile flavor substances in pectoral muscles was detected by using headspace solid-phase microextraction combined with comprehensive two-dimensional gas chromatography-time-of-flight mass spectrometry. The lipid composition of pectoral muscles was determined by liquid chromatography-mass spectrometry, and the differentially expressed genes in duck pectoral muscles were determined by a high-throughput sequencer. Through integrated analysis, the main substances and main genes that affect the flavor of ducks were identified. The results showed that the detection results of flavor substances indicated that eight differential volatile compounds were screened out in the comparison between WQ ducks and CV ducks, namely, 4(1H)-Pyridinone, 2, 3-dihydro-1-methyl-, 1-Octen-3-ol, Hexanoic acid, trans-4-tert-butylcycloheptanol, 1-Octanol, 2, 4-Decadienal, (E, E)-, N, N′-Diacetylethylenediamine, Pyrimidine, 4-butyl-3, 4-dihydro-5-methyl-. The differential volatile compounds were mainly manifested in hydrocarbons, aldehydes and ketones, esters. A total of 86 differential lipids were screened out in the comparison between WQ ducks and CV ducks. Among them, the lipid differences between WQ and CV ducks were mainly in triglycerides (TG), phosphatidylcholines (PC) and glycerophospholipids (PE). A total of 500 differentially expressed genes were screened out in the comparison between WQ ducks and CV ducks. These differentially expressed genes were mainly involved in pathways such as glycerolipid metabolism, fatty acid degradation, and regulation of lipolysis in adipocytes. In summary, this study screened and determined that eight volatile organic compounds were the key aroma substances in the pectoral muscles of the two groups of ducks, and 86 differential lipids could distinguish the pectoral muscles of the two groups of ducks. Triglycerides (TG), phosphatidylcholines (PC) and glycerophospholipids (PE) played a crucial role in the formation of volatile compounds in pectoral muscles. The differentially expressed genes *TPP1* and *PNPLA2* might play an important role in the generation of flavor substances in duck pectoral muscle tissues.

## 1 Introduction

With the improvement of people’s living standards, meat quality has become an important goal in animal breeding. Meat quality is a complex trait and is comprehensively evaluated through a series of indicators such as meat color, intramuscular fat content, pH value, water-holding capacity and tenderness. Flavor is an important component of meat quality (Fu et al., 2022), consisting of non-volatile taste substances and volatile aroma substances (Khan et al., 2015). Among them, volatile compounds are one of the important factors affecting the flavor of meat quality (Domínguez et al., 2014), making different meat qualities present different flavors. The main pathways for the generation of volatile compounds are lipid oxidation and the Maillard reaction (Liu et al., 2019). Therefore, lipids are inextricably linked to the formation of volatile compounds (Resconi et al., 2013) and are also the most important source for meat substances to produce specific flavors (Shahidi and Hossain, 2022).

Wuqin No. 10 duck (WQ ducks) is a newly developed meat duck breed in recent years. It has distinct appearance characteristics, a moderately sized body, good meat flavor and high feed efficiency. Gaining an in-depth understanding of the internal mechanism of flavor substance formation in WQ ducks and exploring potential regulatory methods are of great significance for further improving meat quality in the future. Recently, multi-omics association analysis has become popular in the research on potential molecular mechanisms in the studies of complex traits and diseases (Arora et al., 2021; Cardoso et al., 2021; Lu et al., 2021). Lipidomics (Song et al., 2022) is a research model that can systematically analyze the composition and expression changes of biological lipids (Wenk, 2010). Zheng et al. revealed the lipid patterns and gene expressions of subcutaneous fat in small-sized meat ducks through lipidomics and transcriptomics, laying a foundation for future research on the subcutaneous fat deposition of small-sized meat ducks (Zheng et al., 2025). Tang et al. demonstrated that riboflavin supplementation significantly increased the contents of phosphatidylglycerol and coenzyme Q in the breast meat of Beijing ducks, as well as the levels of two favorable key odorants, namely, citronellyl acetate and 3-(methylthio)propanal through lipidomics and volatile compound analysis (Tang et al., 2023). Flavoromics specifically conducts a comprehensive characterization of the components that affect meat flavor and, combined with techniques such as chemometrics, mines, screens and identifies the key compounds affecting flavor (Ronningen et al., 2018; Yao et al., 2025). These have been proven to be powerful tools for studying the muscle regulatory systems that drive meat quality traits (Wang et al., 2020; Dou et al., 2022).

Therefore, in this study, headspace solid-phase microextraction combined with comprehensive two-dimensional gas chromatography-time-of-flight mass spectrometry, liquid chromatography-mass spectrometry and high-throughput sequencing technology were used to analyze the differences in the lipid and flavor substance compositions between WQ ducks and Cherry Valley ducks (CV ducks), as well as the key genes involved in the lipid composition and flavor substances among duck breeds, in the hope of providing new ideas for the improvement of the meat quality of WQ ducks.

## 2 Materials and methods

### 2.1 Experimental animals and design

CV ducks and WQ ducks were raised under the same standardized management conditions. One hundred and twenty 3-day-old CV ducks and 120 WQ ducks were randomly divided into 12 replicates, and each replicate consisted of 10 ducks (with an equal number of males and females). All ducks were provided with the same uniform feed, which is a commercial feed based on corn and soybeans. The ducks had free access to feed and water, and did not take any antibiotics within 1 month before slaughter. At the age of 63 days, one duck was randomly selected from each replicate and fasted for 8 h (n = 12). After slaughter, the pectoral muscle tissues were immediately collected and stored in liquid nitrogen for subsequent analysis. All procedures involving animals were approved by the Animal Care and Committee of Wuhan Academy of Agricultural Sciences (WHAAEC-2024-018). Sample isolation was carried out in accordance with the Animal Care and Use Statute of China.

### 2.2 Metabolome analysis

Volatile metabolites were extracted from 1 g tissue samples via automated headspace solid-phase microextraction (HS-SPME). Samples were mixed with 1 mL saturated NaCl and 10 μL internal standard solution, then subjected to HS-SPME (Supelco) coupled with GC-MS (Agilent 7890B/5977 A) under optimized conditions: extraction at 60°C for 20 min, desorption at 250°C for 5 min, and chromatographic separation on a DB-WAX column (30 m × 0.25 mm × 0.25 μm) with helium carrier gas (1.2 mL/min). Mass spectra were acquired in electron ionization mode (70 eV, m/z 35-450). Raw data were processed using MassHunter (Agilent) for peak extraction, retention index calculation (n-alkane standards), and filtering of peaks with >50% missing values. Internal standard-normalized peak areas were log_2_-transformed for statistical analysis.

### 2.3 Lipidome analysis

Lipids were extracted using the Folch method (Mopuri et al., 2020) (chloroform:methethanol 2:1, v/v) and fractionated by solid-phase extraction (SPE) on silica columns. Non-polar impurities were removed with n-hexane, followed by lipid elution with chloroform:methanol (9:1, v/v). Lipid profiling was performed via LC-MS/MS using Cortecs C18 (positive mode) and XSelect CSH C18 columns (negative mode) with mobile phases A (acetonitrile/water, 60:40, 10 mM ammonium acetate) and B (isopropanol/acetonitrile, 90:10). A 35-min gradient (30%-98% B) was applied, and data were acquired on a Thermo Q-Exactive HF-X in data-dependent acquisition mode (spray voltage: ±3.2/2.8 kV, resolution: 120,000 MS1/15,000 MS2). Lipids were annotated using LipidSearch 3.2 (mass tolerance ≤5 ppm precursor, ≤10 ppm fragment) with retention time deviation <0.25 min and chromatographic area >5 × 10^6^.

### 2.4 Transcriptome sequencing and analysis

Total RNA was extracted from tissues using TRIzol (Invitrogen), with RNA integrity verified (RIN >8.0, Bioanalyzer 2100). mRNA was enriched via oligo-dT beads and sequenced on an Illumina NovaSeq 6000 (PE150). Raw reads were trimmed using Trimmomatic (v0.38) and aligned to the *Anas platyrhynchos* genome (ENSEMBL v103) via HISAT2. Gene expression was quantified with HTSeq (v0.11.3), and differentially expressed genes (DEGs) were identified using DESeq2 (|log2FC| >1, FDR-adjusted *P* < 0.05). Functional annotation of DEGs was performed against GO and KEGG databases, with enriched pathways (Fisher’s exact test, FDR <0.05) linked to lipid metabolism and flavor compound synthesis.

### 2.5 Verification of the accuracy of transcriptome data by RT-qPCR

In this study, RT-qPCR was used to verify the accuracy of the transcriptome sequencing data. First, six genes were randomly selected from the differentially expressed genes, including four upregulated genes and two downregulated genes. The mRNA of the above eight genes was used as the template for RT-qPCR analysis. The primer information is shown in Supplementary Table S1. The specific method of RT-qPCR refers to Xu et al. (Xu et al., 2021).

### 2.6 Integrated analysis of transcriptome, metabolome and lipidome

The spearman correlation coefficient was used to evaluate the correlations between differentially expressed genes (DEGs) and volatile compounds (VCs) as well as significantly different lipids (SDLs) respectively. A heatmap was plotted using heatmap tools in the genescloud platform (https://www.genescloud.cn). This tool was developed from the pheatmap package (V1.0.8), which was slightly modified to improve the layout style. The data was normalized by z-scores. The package utilizes popular clustering distances and methods implemented in the dist and hclust functions in R.

### 2.7 Data statistical analysis

The data were analyzed by Mann-Whitney U test in SPSS 24.0. *P < 0.05 indicates a significant difference, **P < 0.01 indicates an extremely significant difference.

## 3 Results

### 3.1 Flavor characteristics of CV ducks and WQ ducks

**FIGURE 1:**
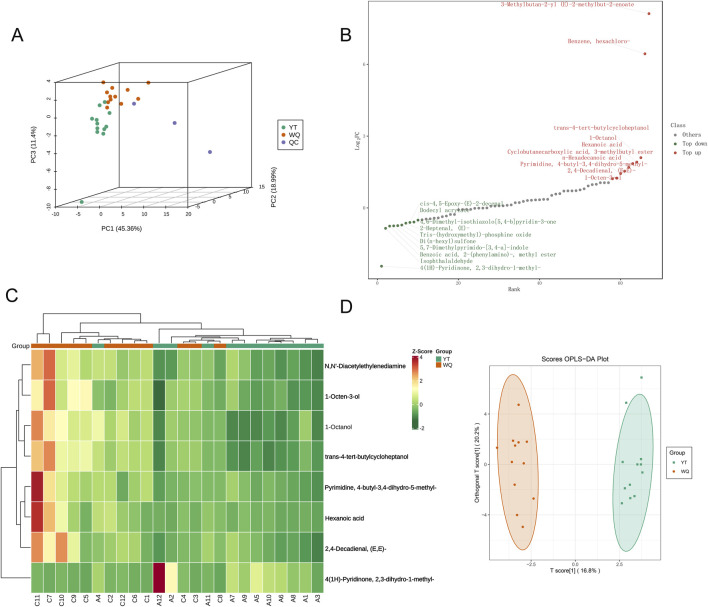
Comparison of volatile metabolomic data between WQ ducks and CV ducks. **(A)** Principal Component Analysis of the Overall Sample. **(B)** Dynamic distribution of SDVs in pectoral muscle between WQ ducks and CV ducks. **(C)** Clustering heatmap of SDVs in pectoral muscle between WQ ducks and CV ducks (Note: A represents CV duck, C represents WQ duck). **(D)** Orthogonal Partial Least Squares-Discriminant Analysis.

Twenty - four samples from the pectoral muscle tissues of WQ and CV ducks were subjected to flavor characteristic analysis using headspace SPME-GC- MS). A total of 67 VCs belonging to 11 different chemical families were identified in the samples. These compounds included 6 hydrocarbons, 8 alcohols, 20 aldehydes, 7 ketones, 3 acids, 14 esters, 1 ether, 4 nitrogen - containing compounds, 2 sulfur - containing compounds and 2 other heterocyclic compounds (Supplementary Table S2). In terms of relative content, aldehydes, alcohols, and esters were the most abundant volatile compounds in the pectoral muscle tissues. The results showed that the relative abundances of aldehydes, acids, ethers, and sulfur compounds in the pectoral muscle tissues of WQ ducks were significantly higher than those of CV ducks. These results emphasized the significant impact of breed on the volatile metabolites in the pectoral muscle tissues of ducks. Principal component analysis (PCA) of all samples showed that the samples of WQ and CV ducks exhibited a separation trend (Figure 1A). To further analyze the differences in the volatile metabolomics of different breeds, a screening criterion (|log_2_ (FoldChange)| ≥ 1; VIP value ≥1; *P* ≤ 0.05) was set to screen for significantly different volatile metabolites (SDVs). The bar chart showed that there were 7 SDVs that significantly increased and 1 SDV that significantly decreased in the pectoral muscles of WQ ducks compared with CV ducks (Figure 1B). To facilitate the observation of the variation patterns of the relative contents of metabolites, we performed row-wise normalization (Unit Variance Scaling, UV Scaling) on the original relative contents of the differential metabolites identified using the screening criteria. A heatmap was plotted using the ComplexHeatmap package in R software. The results showed that the differential metabolites were more enriched in WQ ducks (Figure 1C). Orthogonal partial least squares discriminant analysis (OPLS-DA), which combines the orthogonal signal correction (OSC) and PLS-DA methods, can decompose the information in the X matrix into two types of information related and unrelated to Y. By removing the unrelated differences, differential variables can be screened. The results showed that the samples in the CV duck might gather in one area of the score plot, while the samples in the WQ duck gathered in another area, and there was a clear separation between the two areas, indicating that the model could well distinguish the two groups of samples (Figure 1D).

### 3.2 Comparison of lipidomic profiles between the CV ducks and WQ ducks

**FIGURE 2:**
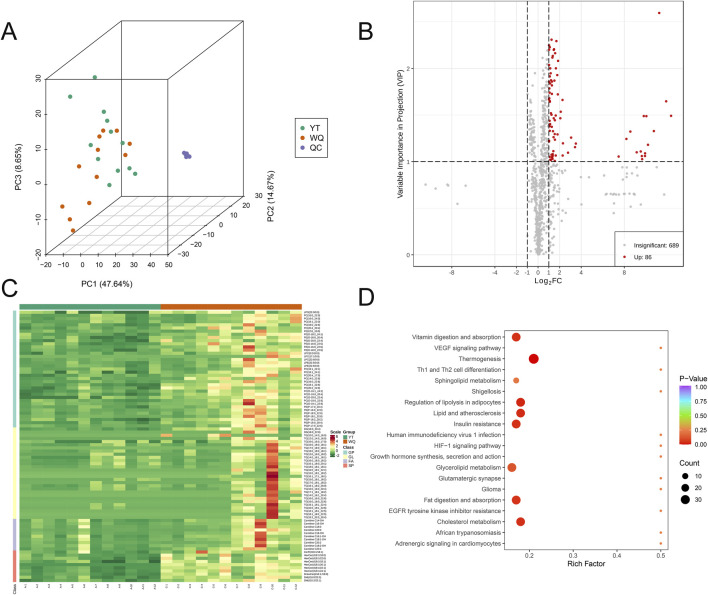
Comparison of lipidomics data between WQ ducks and CV ducks. **(A)** Principal Component Analysis of the Overall Sample. **(B)** Volcano plot of SDLs in pectoral muscle between WQ ducks and CV ducks. **(C)** Heatmap of SDLs in pectoral muscle between WQ ducks and CV ducks (Note: A represents CV duck, C represents WQ duck). **(D)** KEGG enrichment analysis of SDLs in pectoral muscle of WQ ducks and CV ducks.

The flavor of meat is closely related to its lipids. Therefore, we conducted a lipidomics analysis on all samples to analyze their lipid characteristics. In terms of lipid content, triglycerides (TG), phosphatidylcholine (PC), and phosphatidyl ethanolamine (PE) had the highest proportions in the pectoral muscle, accounting for approximately 64.70% of the total lipids (Supplementary Table S3). In the pectoral muscle, the breed had a major impact on the proportions of phosphatidylcholine (PC), sphingomyelin (SM), phosphatidyl ethanolamine (PE), and glycosphingolipids (HexCer) (Supplementary Table S3). Principal component analysis (PCA) showed a separation trend among the groups, suggesting that there were differences in the lipidomes among the sample groups (Figure 2A). A screening criterion (|log_2_(FoldChange)| ≥ 1; VIP value >1; *P* < 0.05) was set to screen for significantly different lipid metabolites (SDLs). The volcano plot showed that compared with CV ducks, there were only 86 SDLs that significantly increased in the pectoral muscles of WQ ducks, and there were no SDLs that significantly decreased (Figure 2B). Among these increased SDLs, TG was predominant. Through the heatmap, to facilitate the observation of the variation patterns of the relative contents of metabolites, we performed row wise normalization (Unit Variance Scaling, UV Scaling) on the original relative contents of the differential metabolites identified using the screening criteria and drew a heatmap using R software. The results showed that more differential lipid substances were enriched in WQ ducks (Figure 2C). The top 50 differential lipids were presented through violin plots (Supplementary Figure S1). Based on the results of the differential metabolites, KEGG pathway enrichment was carried out. Among them, the Rich factor was the ratio of the number of differentially expressed metabolites in the corresponding pathway to the total number of metabolites annotated and detected in that pathway. The larger the value, the greater the degree of enrichment. The results showed that the differential lipids were mainly enriched in signaling pathways such as Vitamin digestion and absorption, Lipid and atherosclerosis, and Regulation of lipolysis in adipocytes (Figure 2D). The combined results indicated that the lipid profiles of duck pectoral muscles showed significant differences among different breeds, which corresponded to the flavor variations in the pectoral muscles.

### 3.3 Correlation between volatile components and lipids

**FIGURE 3:**
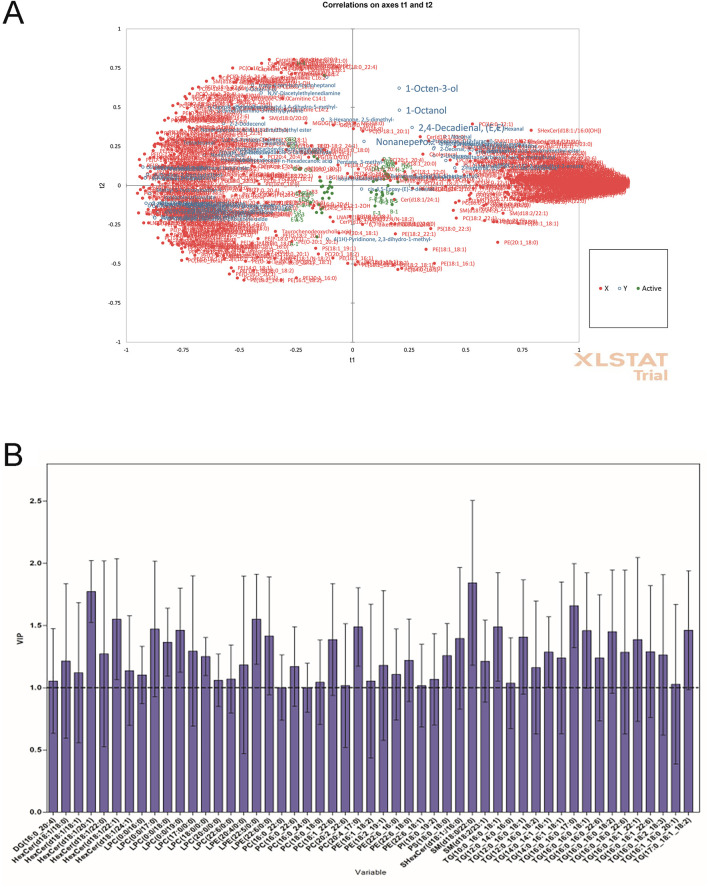
Correlation analysis between metabolome and lipidomics between WQ ducks and CV ducks. **(A)** Correlation loadings plot of the partial least squares regression (PLS-R) analysis between SDLs (X variables) and SDVs (Y variables) in pectoral muscle. **(B)** Potentially important SDLs with variable importance in projection (VIP) scores >1 based on the PLS-R model.

To explore the reasons related to lipid metabolism for the flavor variation of pectoral muscle samples of different WQ and CV ducks, we analyzed the correlation between differential lipids and differential metabolites. The results showed that flavor substances 1-Octen-3-ol (MW0014), 1-Octanol (MW0026) and 2, 4-Decadienal, (E, E)- (MW0039) in the pectoral muscle samples of WQ ducks had a strong positive correlation with lipid substances PC (16:0_22:1), Cer (d18:1/23:0) and SHexCer (d18:1:/16:0(OH)) (Figure 3A). (Figure 3A). We further calculated the VIP (Variable Importance in Projection) values of all SDLs using the PLS-R model. The VIP values of 51 SDLs were ≥1, indicating that they were significant and might contribute to the differences in flavor substances between samples of different varieties (Figure 3B). These results highlight the importance of intramuscular fat in the formation of characteristic flavor compounds in duck meat.

### 3.4 Verification of transcriptome accuracy and analysis of DEGs

The read count of the transcriptome data and the relative expression results of RT-qPCR are shown in Supplementary Figure S2. The expression of the six genes identified by RT-qPCR was consistent with the read count of the transcriptome data, and the fold changes were basically the same. The consistency between RT-qPCR and the transcriptome data indicated the accuracy of the transcriptome sequencing results. By comparing the expression levels of all genes in the WQ group and the CV group, a total of 500 differentially expressed genes (DEGs) were screened out. Specifically, compared with CV ducks, there were 312 DEGs with upregulated expression and 188 DEGs with downregulated expression in the pectoral muscle tissues of Wuqin 10 meat ducks (Supplementary Figure S3A). In addition, among the upregulated genes, we found that some genes, such as *PNPLA2* (Patatin-like phospholipase domain containing 2, which is directly involved in lipid metabolism and regulates lipid storage and energy release.) and *TPP1* (Tripeptidyl peptidase 1, which is related to protein decomposition) may be related to the metabolism of flavor substances and lipid metabolism.

### 3.5 GO functional enrichment and KEGG functional annotation analysis of DEGs

**FIGURE 4:**
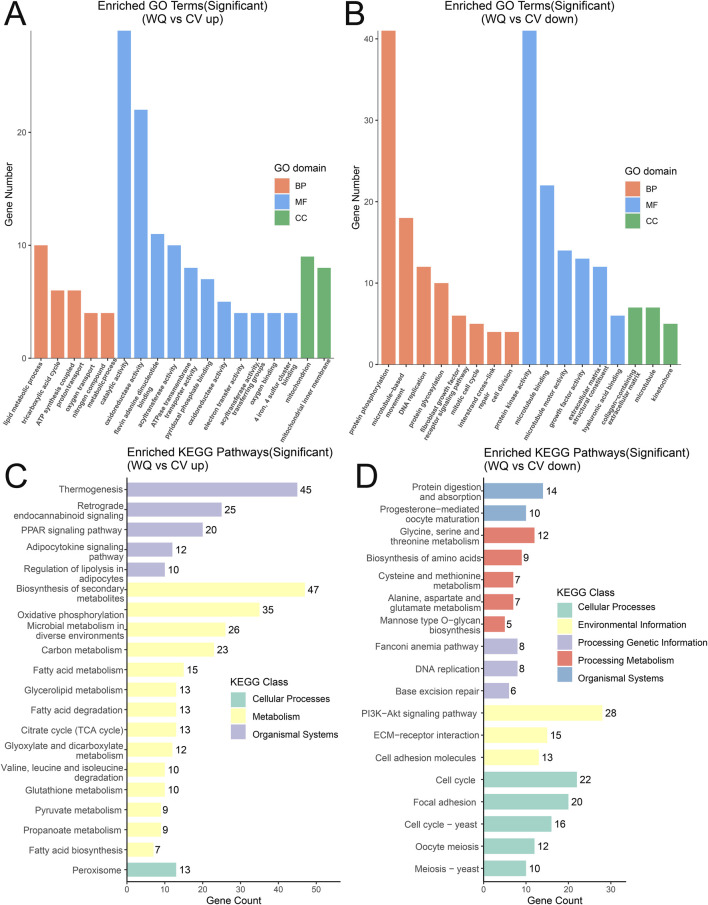
GO enrichment analysis and KEGG functional annotation analysis of DEGs **(A)** GO enrichment analysis of upregulated genes. **(B)** GO enrichment analysis of downregulated genes. **(C)** KEGG functional annotation analysis of upregulated genes. **(D)** KEGG functional annotation analysis of downregulated genes.

To further determine the functions related to the differentially expressed genes, the differentially expressed genes were classified into relevant functional categories through GO annotation, mainly including three categories such as Molecular Function (MF), Biological Process (BP), and Cellular Component (CC) (Supplementary Figure S3B). Among them, the main relevant functions in the MF category were phosphoric diester hydrolase activity, triglyceride lipase activity, phospholipase activity, and lipid binding; the main relevant function in the BP category was lipid metabolic process; and the main relevant function in the CC category was plasma membrane. In addition, the upregulated genes in WQ ducks are mainly involved in lipid metabolic processes, catalytic activity, and mitochondrion; and its downregulated genes are mainly involved in protein phosphorylation, protein kinase activity, and microtubules compared with CV ducks (Figures 4A,B). The results of KEGG pathway annotation indicated that the differentially expressed genes were mainly involved in pathways such as metabolic pathways, neuroactive ligand-receptor interaction, biosynthesis of secondary metabolites, phagosome, and axon guidance. Compared with CV ducks, WQ ducks were significantly enriched in lipid metabolism-related pathways, including the PPAR signaling pathway, glycerolipid metabolism, fatty acid degradation, and regulation of lipolysis in adipocytes (Supplementary Figure S3C). In addition, the upregulated genes of WQ ducks are mainly involved in the pathways of fatty acid biosynthesis, fatty acid metabolism, and regulation of lipolysis in adipocytes. In contrast, the downregulated genes are mainly involved in the pathways of protein digestion and absorption, PI3K-AKT signaling pathway, and glycine, serine and threonine metabolism compared with CV ducks (Figures 4C,D).

### 3.6 Integrated analysis of transcriptome, metabolome and lipidome

**FIGURE 5:**
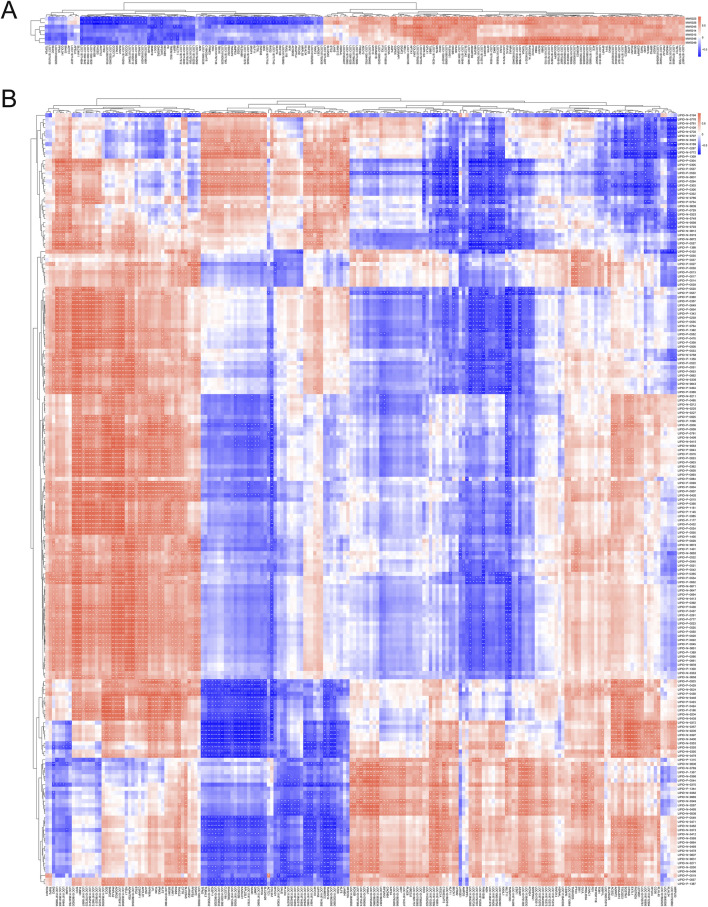
Correlation analysis between DEGs and SDVs as well as SDLs respectively. **(A)** Correlation analysis between DEGs and SDVs. **(B)** Correlation analysis between DEGs and SDLs.

To explore the most crucial genes affecting duck flavor, we conducted correlation analyses between significantly different metabolites and lipids and differentially expressed genes respectively. Each row represents a metabolite, and each column represents a gene. Nonaneperoxoic acid, 1, 1 - dimethylethyl ester (MW0045) and 2, 4 -Decadienal, (E, E)- (MW0046) showed significant positive correlations with *LOC101797323*, *LOC119718680*, *LOC113839608*, *LOC101791103*, *TPP1* and *PNPLA2* (*P* < 0.05). These two metabolites had significant negative correlations with *SMIM19*, *ODC1*, *MLLT11*, *LOC119717912*, *BBIP1*, *LOC119714654*, *CHAD*, *TMEM150C*, *PPDPFL* and *MLLT11* (*P* < 0.01 or *P* < 0.05) (Figure 5A; Supplementary Table S4). In addition, the *TPP1* and *PNPLA2* showed significant positive correlations with TG (16:0_16:0_22:6) (LIPID-P-1146), LPC (0:0/17:0) (LIPID-P-0355), TG (17:0_18:1_18:2) (LIPID-P-0985), TG (16:0_18:1_22:6) (LIPID-P-1177), and TG (16:0_18:1_22:1) (LIPID- P-0935) respectively (P < 0.05). The differentially expressed genes *MLLT11*, *TMEM150C*, *PPDPFL* and *MLLT11* showed significant negative correlations with (LIPID-P-1146), (LIPID-P-0355), (LIPID-P-0985), (LIPID -P-1177) and (LIPID -P-0935) respectively (*P* < 0.01 or *P* < 0.05) (Figure 5B; Supplementary Table S4).

## 4 Discussion

The flavor of meat products is formed by the combined action of a variety of compounds generated through a series of changes (such as lipid oxidation reactions, Maillard reactions, etc.) of precursors (Sun et al., 2022). Currently, there are more than a thousand kinds of volatile flavor substances detected in meat products, and the composition and content of these flavor substances affect the aroma and taste of meat to a certain extent. According to the screening of the main aroma contributors by Relative Odor Activity Value (ROAV), it was found that the differences in the contents of aldehydes and ketones might be the reasons for the flavor differences in duck meat. Among them, aldehydes are mostly produced by fat oxidation, have a low threshold, and contribute significantly to the flavor of meat (Liu D. et al., 2022). They are the most important class of compounds in the flavor components of meat. Hexanal is a relatively important aldehyde in duck meat (Rannou et al., 2013). Moreover, in this study, the relative content of hexanal in the muscle tissues of WQ ducks was significantly higher than that of CV ducks. Hexanal has flavor characteristics such as a grassy flavor, a fatty flavor, and a raw fishy flavor (Xu et al., 2024). In duck meat, this flavor can be regarded as part of the unique flavor of duck meat to a certain extent. The lipids in duck meat, especially unsaturated fatty acids, are the main sources for the production of hexanal during the oxidation process. When duck meat is exposed to air or affected by some oxidation conditions during the processing, unsaturated fatty acids will undergo oxidation reactions. This process is a free radical chain reaction. Firstly, the double bond positions of unsaturated fatty acids are attacked by free radicals, and then, after a series of oxidation and decomposition steps, aldehydes such as hexanal are finally generated.

In this study, it was detected that 2, 4-Decadienal, (E, E)- had a relatively high odor activity in the pectoral muscle tissues of WQ ducks. 2, 4-Decadienal, (E, E)- has typical fatty and fried aromas (Zhang et al., 2018). At low concentrations, this aroma can add a pleasant fragrance to the muscle and is one of the important components that constitute the characteristic aroma of meat, indicating that 2, 4-Decadienal, (E, E)- makes an important contribution to the aroma of duck meat. Ketones are a representative class of products of fat oxidation and Maillard reactions. They make the flavor of meat more layered and play an important role in the composition of the meat aroma (Kao et al., 2012). Because they also have fruity and creamy aromas, they have a relatively positive impact on the flavor. Studies by Legako et al. (Legako et al., 2018) have found that 2,3-butanedione and 3-hydroxy-2-butanone may exist in raw beef and are usually regarded as components of the Maillard reaction. In this experiment, the relative content of ketone compounds in the muscle tissues of WQ ducks was significantly higher than that of CV ducks, indicating the important contribution of ketone compounds to the volatile flavor.

The content of furan compounds in the pectoral muscles of the WQ ducks was higher than that in the CV ducks. Furan compounds are usually regarded as the main aroma components of meat (Meng et al., 2017; Yeo et al., 2022). They mainly come from the enolization and dehydration reactions of carbohydrates (Sung, 2013). Generally, they impart a caramel aroma to meat. Due to their low threshold, furan compounds contribute greatly to the meat aroma of WQ ducks.

The flavors of meat products of different breeds vary, and lipid reactions are usually considered to be the main source of the characteristic flavors of meat products. Some studies have shown that the aroma differences among lambs with different intramuscular fat contents may be related to the thermal oxidation of oleic acid, linoleic acid and linolenic acid as well as the release rate of aroma compounds (Liu H. et al., 2022). Some studies have analyzed the odors of native Chinese pigs and found that 15 volatile flavor substances are the characteristic substances of native pigs, and fatty acids such as oleic acid, docosahexaenoic acid and α-linolenic acid are the main precursors for local pig breeds to form rich flavors (Wu W. et al., 2022). Currently, more and more studies are using lipidomics techniques to analyze the lipid components in meat products and then explore the relationship between lipids and volatile flavor substances. However, studies on analyzing the lipid composition in halogenated duck meat products using lipidomics are rarely reported. In this study, lipidomics analysis was conducted on CV ducks and WQ ducks. A total of 86 different lipid classes were identified in this study, among which PC and TG were in relatively large numbers and mainly belonged to GP and GL, which was consistent with the research results of Nazari and Muddiman (Nazari and Muddiman, 2016) and Mi et al. (2018). In this study, PC accounted for the highest proportion in GP, which was consistent with the result shown in most studies that PC is the most abundant glycerophospholipid in the skeletal muscle cells of most animals (Li et al., 2020). Man et al. (2023) found that PC plays a key role in the formation of volatile compounds in meat. Zhang et al. (2022) indicated that phospholipids may be important factors affecting muscle development and chicken quality. Phospholipid hydrolysis first generates fatty acids, which are then further oxidized to produce aldehydes and ketones. Among them, C18: 2 and C20: 4 can be degraded to form hexanal and nonanal respectively. The differences in the types and contents of PC and PE rich in unsaturated fatty acids in different breeds of duck meat may be the reasons for the differences in the characteristic volatile flavor substances between CV and WQ ducks. Therefore, it is speculated that PC and PE may play a key role in the formation of volatile flavor substances in meat. Further analysis found that unsaturated fatty acids with more than 18 carbon atoms may be the main pathway for lipids to degrade and generate volatile flavors. This result further clarifies the main pathways and key substances for lipid reactions to form volatile flavor substances, but the specific transformation methods still need in-depth research.

The results of the integrated analysis revealed that *TPP1* and *PNPLA2* had a significantly positive correlation with the main VCs and SDLs, and they might have a potential role in meat quality flavor. Protein degradation products (such as peptides and amino acids) are important precursors for the generation of flavor substances (Li et al., 2022; Smit et al., 2005). The peptides and free amino acids produced by Tripeptidyl peptidase one can generate flavor substances through the Maillard reaction with reducing sugars (Pei-Ge et al., 2006; Wang et al., 2019). For example, during the cooking process, these amino acids and peptides undergo the Maillard reaction with reducing sugars to produce volatile flavor substances such as pyrazines, aldehydes, and ketones with flavors such as roasted and nutty aromas, thus affecting the flavor of the meat. Patatin -like phospholipase domain-containing 2 has phospholipase activity and might be involved in the catabolism of intramuscular fat in muscle cells (Schrammel et al., 2013; Yang and Mottillo, 2020). During the maturation and processing of meat, the hydrolysis and oxidation of fat are one of the important pathways for the generation of flavor substances (Christlbauer and Schieberle, 2009; Wu H. et al., 2022; Wu et al., 2024). If the activity of this protein is enhanced, it might promote more fat to be decomposed into fatty acids and glycerol, thereby providing more precursor substances for the subsequent generation of flavor substances.

## 5 Conclusion

This study utilized metabolomics, lipidomics, and transcriptomics to identify eight differentially expressed volatile organic compounds, 86 differentially expressed lipids, and 500 differentially expressed genes in two groups of duck pectoral muscles, respectively. Determine 1-Octen-3-ol (MW0014), 1-Octanol (MW0026), 2,4-Decadienal, (E, E) - (MW0039), Nonaneperoxoic acid, 1,1-diethylethyl ester (MW0045), and 2,4-Decadienal, Volatile organic compounds such as (E, E) - (MW0046) are key aroma compounds in the pectoral muscle of WQ ducks, contributing to the flavor of duck meat. These results provide theoretical basis and data support for the development of duck germplasm resources and the evaluation of local duck flavor quality.

## Data Availability

The original contributions presented in the study are publicly available. This data can be found here: https://www.ncbi.nlm.nih.gov/bioproject/?term=PRJNA1262909.

## Supplementary material

SUPPLEMENTARY FIGURE S1 The top 50 SDLs.

SUPPLEMENTARY FIGURE S2 Verification of transcriptome accuracy.

SUPPLEMENTARY FIGURE S3 Comparison of transcriptome data between WQ ducks and CV ducks. **(A)** Volcano plot of DEGs in WQ ducks compared to CV ducks. **(B)** GO enrichment analysis of DEGs; **(C)** KEGG enrichment analysis of DEGs.

## Abbreviations

WQ ducks, Wuqin 10 ducks; CV ducks, Cherry Valley ducks; TG, Triglycerides; PC, Phosphatidylcholines; PE, Glycerophospholipids; HS-SPME, Headspace solid-phase microextraction; GC-MS, Gas Chromatography-Mass Spectrometry; SPE, Solid-phase extraction; GO, Gene Ontology; KEGG, Kyoto Encyclopedia of Genes and Genomes; DEGs, Differentially expressed genes; VCs, Volatile compounds; SDLs, Significantly different lipids; PCA, Principal component analysis; SDVs, Significantly different volatile metabolites; UV Scaling, Unit Variance Scaling; OPLS-DA, Orthogonal partial least squares discriminant analysis; OSC, Orthogonal signal correction; SM, Sphingomyelin; HexCer, Glycosphingolipids; MF, Molecular Function; BP, Biological Process; CC, Cellular Component.
