# Supplementary material for: Multi-omics analysis of flavor differences in pectoral muscles between Wuqin 10 duck and Cherry valley duck
Source: Front Mol Biosci. 2025 May 30;12:1558907. doi: 10.3389/fmolb.2025.1558907 (PMC12162291; doi:10.3389/fmolb.2025.1558907)
Supplement: Supplementary file 3 [file Table1.docx]

Supplementary Table S1. The primers for qRT-PCR.

| Gene | Primer sequences (5**’**→3’) | Product length, bp |
| --- | --- | --- |
| *CPLX1* | F: CCTTTGCACAGCCACACAAG  R: CCCCAGGCAAAGTTCAACAC | 276 |
| *BDH1* | F: ATCCAAGGACTCAAGCCAGC  R: GTCAGTGGTGACCCTCACAG | 204 |
| *TPP1* | F: CTCGGACAACTACTGGGTGG  R: CCCTGCGTCACATCGTAGAG | 210 |
| *GATA3* | F: TTCTCTGCCCTTTCCCAACC  R: CTCTGCGAAGTGAGATCCCC | 295 |
| *NECAB3* | F: CCACTTGCAGTCCAGCTACA  R: ATTTTCTGCCCCGGAGTGAG | 176 |
| *TNNT2* | F: GACCACCTCAGCGAAGACAA  R: GCCTTCGACCCTTTGACCTT | 173 |
| *GAPDH* | F: GGTTGTCTCCTGCGACTTCA  R: TCCTTGGATGCCATGTGGAC | 165 |
