# Supplementary figures and images for: Multi-omics analysis of flavor differences in pectoral muscles between Wuqin 10 duck and Cherry valley duck
Source: Front Mol Biosci. 2025 May 30;12:1558907. doi: 10.3389/fmolb.2025.1558907 (PMC12162291; doi:10.3389/fmolb.2025.1558907)

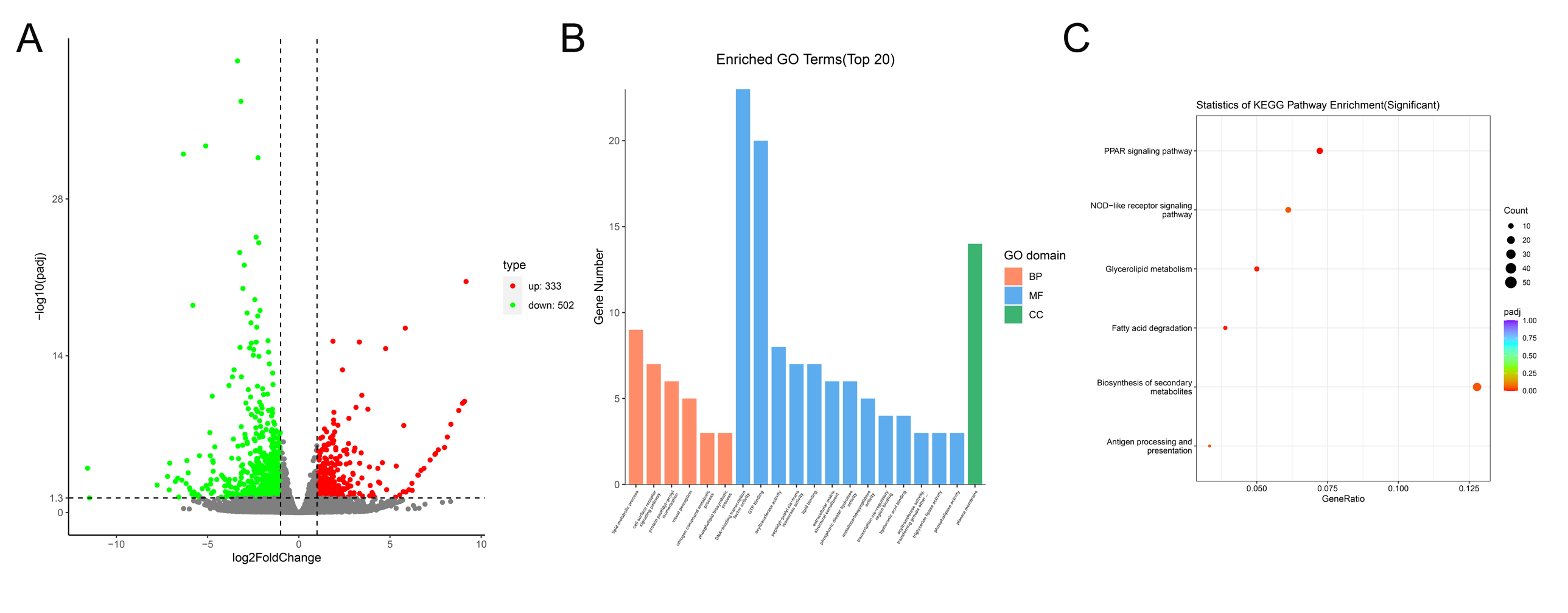

Supplement: Supplementary file 4 [file Image3.tif]

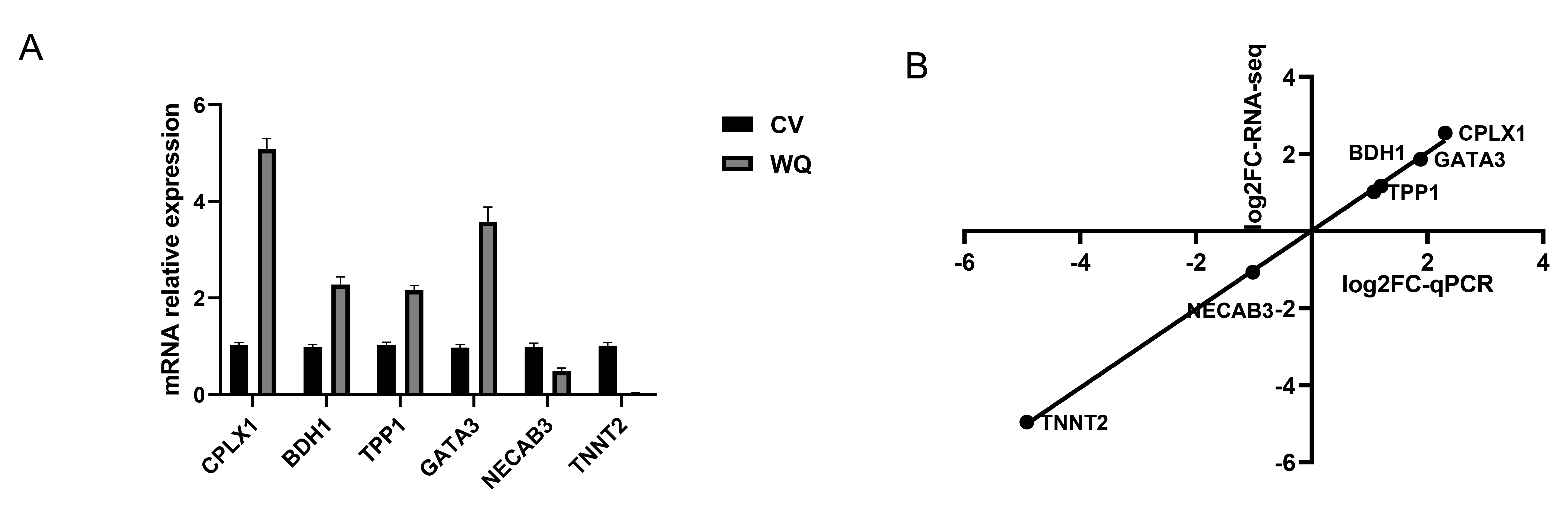

Supplement: Supplementary file 5 [file Image2.tif]

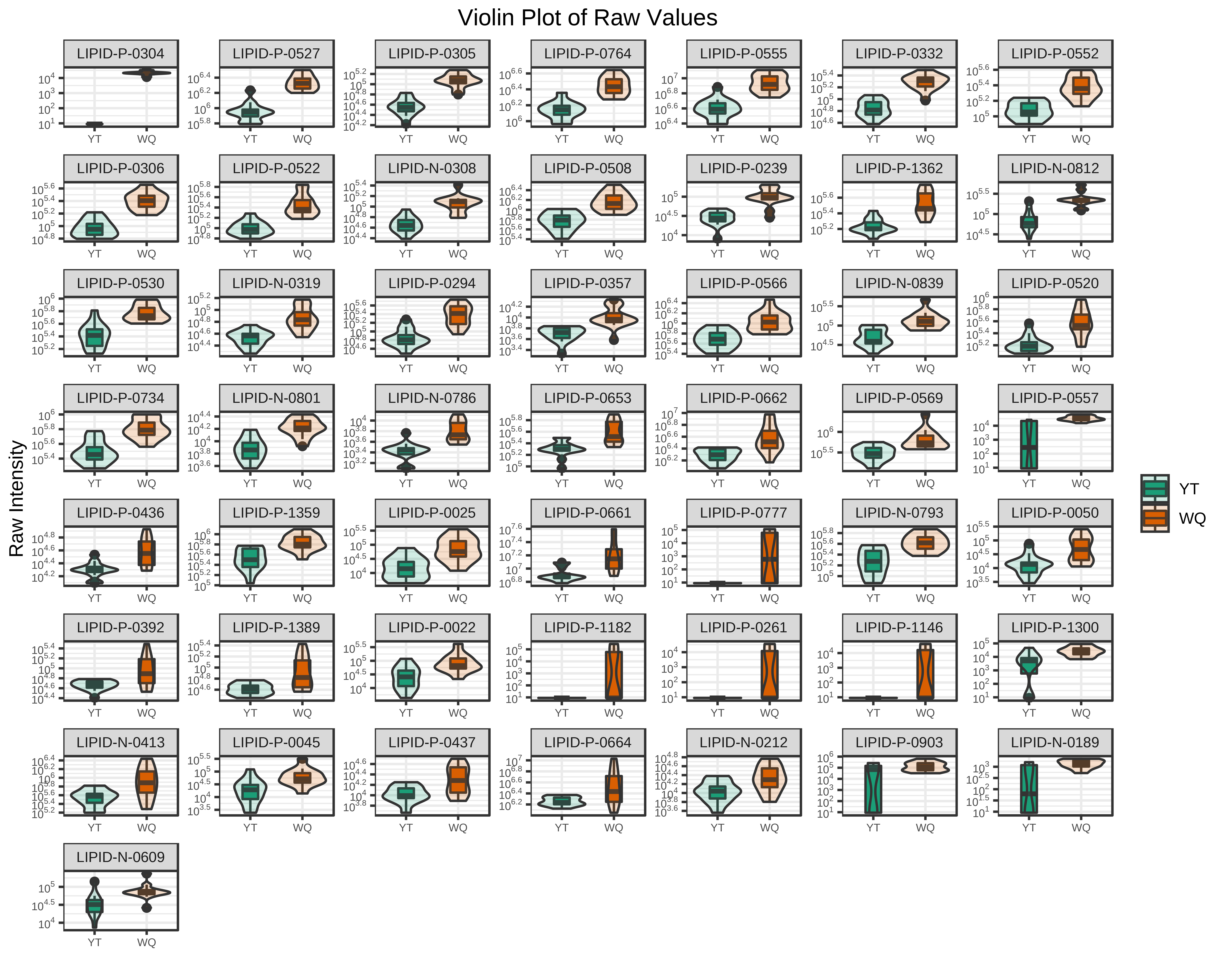

Supplement: Supplementary file 6 [file Image1.tif]
